# Cruciferous vegetables improve glycaemic control compared to root/squash vegetables in a randomized, controlled, crossover trial: The VEgetableS for vaScular hEaLth (VESSEL) study

**DOI:** 10.1111/dom.16467

**Published:** 2025-05-15

**Authors:** Emma L. Connolly, Alex H. Liu, Richard J. Woodman, Armaghan Shafaei, Lisa G. Wood, Richard Mithen, Anthony P. James, Carl J. Schultz, Seng Khee Gan, Catherine P. Bondonno, Joshua R. Lewis, Jonathan M. Hodgson, Lauren C. Blekkenhorst

**Affiliations:** ^1^ Nutrition and Health Innovation Research Institute, School of Medical and Health Sciences Edith Cowan University Joondalup Western Australia Australia; ^2^ Flinders Health and Medical Research Institute Flinders University Adelaide South Australia Australia; ^3^ Centre for Integrative Metabolomics and Computational Biology School of Science, Edith Cowan University Joondalup Western Australia Australia; ^4^ School of Biomedical Science and Pharmacy University of Newcastle Callaghan New South Wales Australia; ^5^ Liggins Institute University of Auckland Auckland New Zealand; ^6^ Curtin School of Population Health Curtin University Perth Western Australia Australia; ^7^ Medical School University of Western Australia Perth Western Australia Australia; ^8^ Department of Cardiology Royal Perth Hospital Perth Western Australia Australia; ^9^ Department of Endocrinology and Diabetes Royal Perth Hospital Perth Western Australia Australia

**Keywords:** cardiovascular disease, cruciferous vegetables, diabetes, glucosinolates, glycaemic control, postprandial glucose response, randomized controlled trial

## Abstract

**Aims:**

Higher cruciferous vegetable (e.g., broccoli) intake is associated with lower risk of type 2 diabetes and cardiovascular disease, but limited causal evidence exists. We investigated if cruciferous vegetable intake improved glycaemic control compared to root/squash vegetables in non‐diabetic adults with elevated blood pressure.

**Materials and Methods:**

This randomized, controlled, crossover trial consisted of two 2‐week dietary interventions (300 g/day cruciferous [active] and root/squash [control] soups with standardized lunch/dinner meals) separated by a 2‐week washout. Participants were blinded to the intervention allocation. Glycaemic measures were a pre‐specified secondary outcome. Flash glucose monitoring measured interstitial glucose every 15‐min throughout both interventions. Mealtimes and consumption were recorded in food diaries. Differences in continuous glucose, glycaemic variability (coefficient of variation [CV]), and overall, lunch, and dinner postprandial glucose response (PPGR; 2‐h mean glucose [PPGR 2‐h] and area under the curve [AUC]) were assessed using linear mixed‐effects regression.

**Results:**

Eighteen participants (female = 89%) completed the study (median [IQR] age: 68 [66–70 years]). Glycaemic variability was lower in the active versus control (mean difference: −2.0%, 95% CI −2.8, −1.1, *p* < 0.001). Overall PPGR 2‐h and AUC were lower in the active versus control (mean difference: −0.14 mmol/L, 95% CI −0.24, −0.04, *p* = 0.005 and −20.1 mmol/L × min, 95% CI −34.1, −6.1, *p* = 0.005, respectively), driven by the dinner PPGR (*p* = 0.004 and *p* = 0.003, respectively). There was no difference in mean continuous glucose for active versus control (*p* = 0.411).

**Conclusions:**

Cruciferous vegetable consumption improved postprandial glycaemic control compared with root/squash vegetables. The clinical impact remains uncertain and warrants further investigation, particularly in individuals with impaired glycaemic control.

**Clinical Trial Registry:**

This trial was registered at www.anzctr.org.au (ACTRN12619001294145).

**Plain Language Summary**

**What is the context and purpose of this research study?**

Observational cohort studies suggest the type of vegetable consumed is important for lowering the risk of type 2 diabetes and cardiovascular disease, but limited evidence exists from randomized controlled trials. We aimed to evaluate the effect of daily consumption of cruciferous vegetables on glycaemic control and stability in comparison to root/squash vegetables in adults with elevated blood pressure and without diabetes.

**What was done?**

This randomized, controlled, crossover trial included two 2‐week dietary intervention periods during which participants consumed vegetable soups (active: cruciferous vegetables and control: root/squash vegetables) twice daily.

**What were the main results?**

Consumption of cruciferous vegetables resulted in lower glycaemic variability and improved postprandial glucose control in comparison to root/squash vegetables.

**What is the originality and relevance of this study?**

This is the first study to compare the relative contribution of vegetable types (cruciferous vegetables versus root/squash vegetables) through the diet on glycaemic outcomes in adults without diabetes. Increasing cruciferous vegetables within a healthy well‐balanced diet may reduce the risk of type 2 diabetes and cardiovascular disease.

## 1 INTRODUCTION

Poor glycaemic control was estimated to occur in 541 million individuals worldwide.^1^ Dysregulated glucose control increases the risk of type 2 diabetes^2^ and subsequent cardiovascular disease (CVD),^3^ and has a substantial societal impact. The total cost attributed to type 2 diabetes with CVD is projected to exceed AUD$18.66 billion in Australia alone by 2031.^4^ Targeting glycaemic control will reduce this burden worldwide. As a leading modifiable risk factor for both type 2 diabetes and CVD, diet is an important target for the prevention and maintenance of these conditions.

High fibre diets (e.g., fruit/vegetables/whole grains) are beneficial for type 2 diabetes prevention.^5^, ^6^, ^7^ Emerging evidence suggests specific vegetables have differing associations with type 2 diabetes risk. Observational studies have highlighted the benefit of cruciferous vegetables (e.g., broccoli).^8^ However, results have been inconsistent, with some suggesting a positive association with diabetes risk.^5^ As observational studies contain inherent methodological weaknesses,^9^ well‐designed randomized controlled trials (RCTs) remain the gold standard in evaluating nutrition evidence.^10^ Available RCT evidence has utilized cruciferous vegetable extracts, or these vegetables in combination with other subtypes, in diabetic populations,^11^, ^12^, ^13^ but there are limited RCTs investigating cruciferous vegetable intake and markers of diabetes risk in populations without diabetes.

We investigated whether cruciferous vegetable consumption, in comparison to root/squash vegetables, resulted in lower mean continuous glucose levels, lower glycaemic variability, and improved postprandial glucose response (PPGR) in otherwise healthy individuals with mildly elevated blood pressure. Glycaemic control was a secondary outcome of the VEgetableS for vaScular hEaLth (VESSEL) Study. This RCT investigated the effects of cruciferous vegetable consumption (active) on CVD risk factors, in comparison to root/squash vegetables (control).^14^ We previously reported that cruciferous vegetable consumption resulted in a 2.5 mmHg and 2.1 mmHg reduction in ambulatory brachial and aortic systolic blood pressure (SBP), respectively, and a 0.2 mmol/L reduction in serum triglycerides compared to the control.^15^

## 2 METHODS

### 2.1 Ethics

The VESSEL Study was approved by the Edith Cowan University Human Research Ethics Committee (2019‐00356‐BLEKKENHORST) and registered at www.anzctr.org.au (ACTRN12619001294145). All participants provided prior written informed consent.

### 2.2 Study design

**FIGURE 1:**
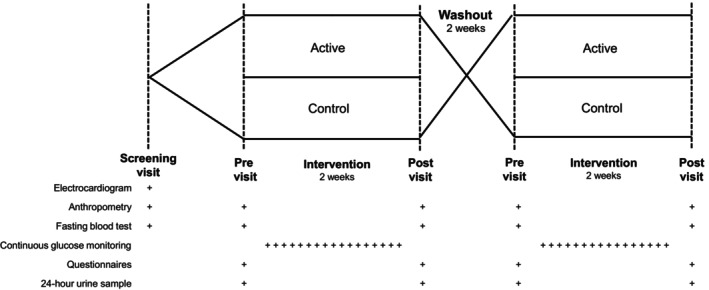
Study design overview.

This was a randomized, controlled, crossover trial including two 2‐week dietary interventions (300 g/day of cruciferous [active] and root/squash [control] vegetable soups) separated by a 2‐week washout period (Figure 1).

### 2.3 Participants

Detailed inclusion/exclusion criteria have been reported previously.^14^, ^15^ Participants included men and women aged 50–75 years located in Perth, Western Australia, with mildly elevated blood pressure (SBP 120‐160 mmHg inclusive and diastolic blood pressure [DBP] <100 mmHg), and who did not have diagnosed diabetes or fasting blood glucose >6.5 mmol/L. Twenty‐one participants were randomly assigned to an intervention sequence order using computer‐generated random numbers (1:1 allocation) concealed in opaque envelopes, with 18 completing the study (Figure S1).^15^

### 2.4 Dietary interventions

In random order, participants completed two dietary interventions: active‐cruciferous vegetables (40% broccoli, 25% cabbage, 25% cauliflower, 10% kale) and control‐root/squash vegetables (20% carrot, 40% potato, 30% pumpkin, 10% sweet potato). Participants were not told which vegetables were included in each intervention or which one was the active/control. Interventions were provided as soups (~600 mL soup/day) containing four vegetable serves daily (~300 g/day). Detailed preparation/storage methods have been reported.^15^ Potato starch was added to the active soup to increase the carbohydrate content, taking into consideration increases in protein and fibre content (active soup: 19 g/day carbohydrate, 11 g/day protein, 0.7 g/day fat, 9 g/day fibre, 589 kJ/day; control soup: 27 g/day carbohydrate, 5 g/day protein, 0.3 g/day fat, 6 g/day fibre, 612 kJ/day). To minimize background diet variation, participants were provided with standard lunch and dinner meals to consume after the soup at lunch and dinner. These meals were frozen, pre‐prepared meals available from the local supermarket, and ingredient lists were checked to ensure no additional cruciferous vegetables were included. Meals were replicated for each individual, for both interventions.^15^

Self‐reported food diaries were completed throughout both interventions. If no mealtime was recorded, this was estimated based on available data. A dietitian (ELC) used the participant's usual timing, food eaten, and glucose data to estimate the mealtime (*n* = 79/1008 [8%] estimated). Participants were instructed to consume breakfast and snacks ad libitum, with no restriction on additional foods consumed. Participants were instructed to avoid consuming any additional foods in the 2‐h window either side of lunch and dinner meals. The nutritional breakdown was determined using Foodworks software, utilizing AusFoods 2019 and AusBrands 2019 databases (Foodworks 10 Professional, v10.0. Brisbane: Xyris Pty Ltd., 2019).

### 2.5 Adherence

Subjective (i.e., self‐reported food diary) and objective (i.e., urinary/plasma S‐methyl cysteine sulfoxide [SMCSO] and serum carotenoids) markers of dietary intake were used to assess intervention adherence. Urine and blood samples were collected at the beginning and end of each intervention for this analysis. Detailed methods have been previously described.^15^

### 2.6 Glycaemic control

Participants were fitted with a flash glucose monitoring system (Freestyle Libre, Abbott Diabetes Care Inc., Alameda, CA, USA) by trained study investigators (AHL, ELC). This was used to measure continuous glucose throughout both interventions. This system has a disposable sensor and an electronic reader. The sensor was applied to the back of the participants' upper arm using the application device and was worn for each 14‐day intervention period. This measures interstitial fluid glucose and stores data every 15 min for an 8‐h period.^14^ Participants were instructed to scan the sensor at least once every 8 h to ensure all recorded glucose data were captured. The reader was covered with opaque black tape so that participants were unable to see their glucose readings. If a participant accidentally removed the sensor, or in the case of a sensor error, the participant was instructed to return to the clinic as soon as possible for a replacement.

Glycaemic control was assessed using differences in continuous glucose, glycaemic variability, and lunch and dinner PPGR. Mean differences in continuous glucose (mmol/L) were assessed using glucose readings aggregated hourly over each intervention period. Glycaemic variability over the two 2‐week interventions was evaluated using the coefficient of variation (%CV) of all stored glucose data.^16^ This was calculated as follows: standard deviation (SD) of all glucose readings divided by mean glucose multiplied by 100. The PPGR after lunch and dinner meals was assessed using (i) 2‐h mean postprandial glucose, and (ii) 2‐h area under the curve (AUC). The glucose reading closest to the reported mealtime was used as timepoint 0 for the determination of both PPGR measurements. Two‐hour mean postprandial glucose and AUC were calculated using the glucose data available every 15 min for the 2‐h period following the mealtime. The trapezoid method was used for the calculation of total AUC. Two‐hour mean postprandial glucose and AUC were calculated for meals overall (i.e., lunch and dinner combined) and for lunch and dinner meals separately.

### 2.7 Lifestyle measures

Weight (kg) was collected using a standardized protocol at the beginning and end of each intervention period.^14^ Physical activity was captured at pre‐ and post‐intervention visits for both interventions using the Community Healthy Activities Model Program for Seniors (CHAMPS)^17^ and energy intake and habitual vegetable intake at baseline were assessed using the Dietary Questionnaire for Epidemiological Studies (DQES v3.2), a validated food frequency questionnaire.^18^ Dietary data were reviewed by a dietitian (ELC) to assess for implausible energy intakes to support body function/lifestyle with respect to BMI, sex, age, and physical activity.^15^

### 2.8 Statistical analysis

#### 2.8.1 Sample size

Sample size estimation has been previously described.^14^, ^15^ Briefly, a desired sample size of 25 participants was calculated to provide greater than 90% power to detect a 0.30 mmol/L difference in 2‐h mean postprandial glucose concentration. This was based on a within‐group SD of 1.5 mmol/L and a minimum of 80 glucose measures for each intervention (2 intervention meals for 14 days, with a measure every 15 min over 2 h), and within‐subject correlation between measures of *r* = 0.6. The calculation utilized the variance inflation factor (VIF) formula for a crossover trial of VIF = N*(1 − *ρ*)/*m*, where *N* is the number of participants needed for a parallel design, *ρ* is the between period correlation (0.6), and *m* = 2 is the number of interventions per subject.^19^

#### 2.8.2 Statistical methods

The normality of continuous variables was assessed using the Shapiro–Wilk normality test. For descriptive statistics, normally distributed variables are presented as mean ± SD, non‐normally distributed variables as median and interquartile range (IQR), and categorical variables as number and proportion (%). Carryover effects were assessed using a paired *t* test comparing fasting serum glucose between intervention arms at each pre‐intervention visit. Fasting serum glucose results have been previously reported.^15^

A modified intention to treat analysis was followed for the primary analyses, including all participants who completed both baseline visits. Per protocol analyses were conducted using only participants with complete data for the outcome being analysed. Participants were required to have at least 70% of possible readings for each 14‐day intervention (i.e., one measurement recorded every 15 min for 14 days) and to have readings on at least 10/14 days^20^ to be included in the per protocol analyses of continuous glucose and glycaemic variability. At least 70% of readings in a 2‐week period have been shown to correlate strongly with the mean glucose level measured over a 3‐month period.^20^

For the per protocol analyses of lunch and dinner PPGR (i.e., 2‐h mean postprandial glucose and 2‐h AUC), a mealtime was not considered valid and was excluded if no intervention soup was consumed. For the AUC analysis, the mealtime was excluded if all 9 readings (0, 15, 30, 45, 60, 75, 90, 105, 120 min) were not recorded. To be included in these analyses, at least 70% of valid postprandial glucose readings were required. Lunch, dinner, and overall 2‐h mean postprandial glucose and AUC were calculated separately.

A linear mixed‐effects regression model was used to test differences in continuous glucose (mmol/L) and glycaemic variability (%CV) over the two 2‐week intervention periods and the lunch and dinner PPGR (i.e., 2‐h mean postprandial glucose and 2‐h AUC). Each model had intervention period entered as a fixed effect and participant ID included as a random intercept. The models for the lunch and dinner PPGR combined also had mealtime included as a fixed effect.

Data were analysed using STATA, version 15.1 (Statacorp, College Station, TX, USA), GraphPad Prism, version 10.0.3 for Mac (GraphPad Software, Boston, MA, USA), and IBM SPSS Statistics for Windows, version 29.0 (IBM Corp., Armonk, NY, USA). Statistical significance was set at a two‐sided Type 1 error rate of *p* < 0.05.

## 3 RESULTS

All 18 participants were included following the intention to treat analysis. For per protocol analyses, one participant was excluded from the analysis of continuous glucose and %CV (*n* = 17 included) and one, three, and two participants were excluded from the analysis of overall (*n* = 17), lunch (*n* = 15), and dinner (*n* = 16) 2‐h mean postprandial glucose and AUC, respectively. Utilizing previously reported data for fasting serum glucose,^15^ no carryover effect between interventions was noted (*p* = 0.846).

### 3.1 Baseline characteristics

Baseline and demographic information are presented in Table 1. Of note, the median age of participants was 68 (IQR 66–70 years) and 89% of participants were female. The mean ± SD fasting blood glucose at screening was 5.5 ± 0.5 mmol/L. The median habitual daily intake of vegetables and cruciferous vegetables was 327.3 g (266.4–367.6 g) and 26.0 g (IQR: 18.5–52.9 g) at baseline, respectively.

**TABLE 1:** Baseline characteristics of all participants.

Characteristic	All participants (*n* = 18)
Age, years	68 (66–70)
Female, *n* (%)	16 (89)
BMI, kg/m^2^	28.1 ± 3.9
Ethnic background, *n* (%)	
Asian	1 (6)
Caucasian	17 (94)
Marital status, *n* (%)	
Single	4 (22.2)
Married	10 (55.6)
De facto	1 (5.6)
Divorced	3 (16.7)
Smoking status, *n* (%)	
Ex‐smoker	7 (39)
Fasting plasma glucose, mmol/L	5.5 ± 0.5
Prediabetes^a^, *n* (%)	3 (16.7)
Triglycerides, mmol/L	1.3 ± 0.5
Total cholesterol, mmol/L	5.5 ± 0.9
LDL cholesterol, mmol/L	3.2 ± 0.5
HDL cholesterol, mmol/L	1.6 (1.3–1.7)
Clinic blood pressure	
SBP, mmHg	135.9 ± 10.0
DBP, mmHg	76.4 ± 7.9
HR, beats/min	69.1 ± 8.8
Energy intake, kcal/day	1804 ± 361^b^
Vegetable intake^c^, g/day	327.3 (266.4–367.6)^b^
Cruciferous vegetable intake, g/day	26.0 (18.5–52.9)^b^

*Note*: Values are presented as mean ± standard deviation, median (interquartile range) or number (percentage) as indicated.

Abbreviations: BMI, body mass index; DBP, diastolic blood pressure; HDL, high‐density lipoprotein; HR, heart rate; LDL, low‐density lipoprotein; SBP, systolic blood pressure.

^a^ Prediabetes was defined as impaired fasting glucose between 6.1 and 6.5 mmol/L based on Australian criteria (6.1–6.9 mmol/L).^44^

^b^ One participant was excluded for implausible energy intake.

^c^ The vegetable variable was calculated according to the Australian dietary guidelines.^45^

### 3.2 Adherence

Urinary and plasma SMCSO were significantly higher following the active compared to the control intervention (*p* < 0.0001 for both) and serum carotenoids (total carotenoids, lutein, lycopene, a‐carotene, and b‐carotene) were significantly higher following the control intervention compared to the active intervention (*p* < 0.05 for all). There was no significant difference in energy expenditure from physical activity between interventions (*p* = 0.600). Participants had significant weight loss from their pre‐ to post‐intervention visits for both interventions (active pre: 72.3 (66.4–84.0 kg) and active post: 71.0 (65.5–82.6 kg) and control pre: 74.3 (66.7–83.2 kg) and control post: 72.4 (64.6–82.0 kg); *p* < 0.001 for both^15^). However, there was no statistically significant difference in weight between interventions (*p* = 0.816). Detailed analysis has been presented previously.^15^

### 3.3 Continuous glucose

**FIGURE 2:**
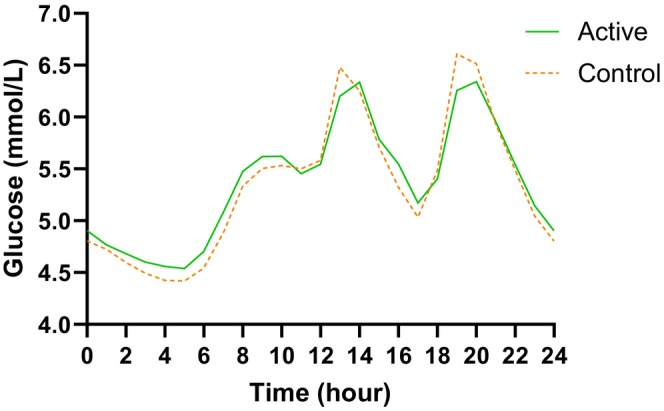
Hourly mean continuous glucose (mmol/L) for both the active and control 2‐week intervention periods.

Figure 2 presents hourly mean continuous glucose (mmol/L) for both intervention periods. Following the intention to treat analysis, the mean ± SD of continuous glucose for the active and control interventions was 5.38 ± 0.79 and 5.34 ± 0.92 mmol/L, respectively. The mean difference in continuous glucose for the active compared to the control intervention was 0.04 mmol/L (95% CI −0.06, 0.15; *p* = 0.411) (Table 2). For the per protocol analysis, the mean difference in continuous glucose for the active intervention compared with the control intervention (mean ± SD active: 5.39 ± 0.80 and control: 5.30 ± 0.91 mmol/L) was 0.09 mmol/L (95% CI −0.01, 0.20; *p* = 0.092) (Table S1).

**TABLE 2:** Measurements of glycaemic control by intervention and between intervention differences following the modified intention to treat analysis.

Outcome	Control	Active	Mean difference active versus control (95% CI)
Continuous glucose, mmol/L	5.34 ± 0.92	5.38 ± 0.79	0.04 (−0.06, 0.15) *p* = 0.411
Glycaemic variability, %CV	20.8 ± 5.8	18.9 ± 5.3	‐2.0 (−2.8, −1.1) *p* < 0.001
2‐h mean glucose^a^, mmol/L			
Overall	6.48 ± 0.81	6.35 ± 0.79	−0.14 (−0.24, −0.04) *p* = 0.005
Lunch	6.47 ± 0.99	6.39 ± 0.89	−0.08 (−0.22, 0.06) *p* = 0.275
Dinner	6.51 ± 0.94	6.33 ± 0.90	−0.20 (−0.33, −0.06) *p* = 0.004
2‐h AUC, mmol/L × min			
Overall	786.57 ± 131.66	768.04 ± 125.05	−20.1 (−34.1, −6.1) *p* = 0.005
Lunch	779.26 ± 144.68	765.53 ± 132.53	−13.29 (−34.97, 8.39) *p* = 0.229
Dinner	793.85 ± 117.14	770.65 ± 117.04	−26.12 (−43.36, −8.89) *p* = 0.003

*Note*: Values are presented as mean ± standard deviation.

Abbreviations: AUC, area under the curve; CI, confidence interval; CV, coefficient of variation.

^a^ Mean glucose value calculated from measurements obtained every 15 min for 2 h following the mealtime.

### 3.4 Glycaemic variability

Following the intention to treat analysis, the mean ± SD of the %CV was 18.9% ± 5.3% and 20.8% ± 5.8% for the active and control interventions, respectively, with a significant difference for the active compared to the control intervention (*D* = −2.0%, 95% CI −2.8, −1.1; *p* < 0.001) (Table 2). For the per protocol analysis, the mean ± SD of the %CV for the active and control interventions was 19.1% ± 5.3% and 21.0% ± 5.8%, respectively, with a significant mean difference active versus control: −1.9%; 95% CI −2.8, −1.1; *p* < 0.001 (Table S1).

### 3.5 Postprandial glucose response

**FIGURE 3:**
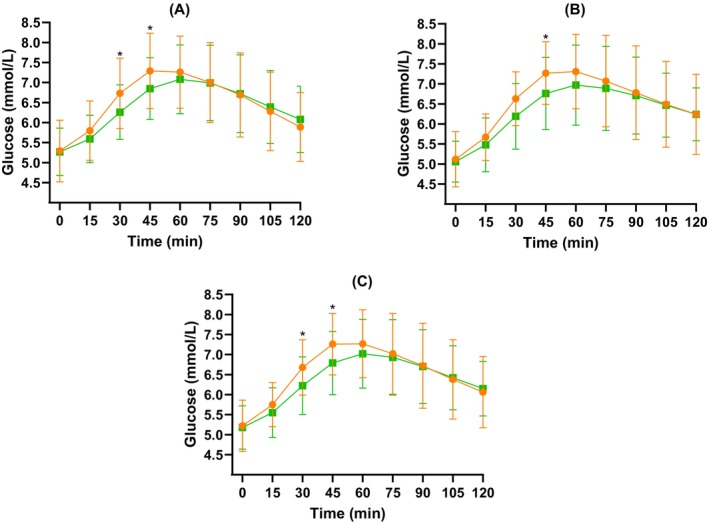
Mean ± standard deviation glucose (mmol/L) at each time point (0–120 min) after (A) lunch, (B) dinner and (C) overall, for both active (green squares) and control (orange circles) interventions. *p < 0.05.

Mean glucose measurements recorded at 15‐min intervals for 2 h following lunch and dinner for each intervention are shown in Figure 3.

#### 3.5.1 2‐h mean postprandial glucose

For the intention to treat analysis, there was a significant difference between interventions for 2‐h mean postprandial glucose for both lunch and dinner meals combined, and for dinner meals alone (mean difference active vs. control: −0.14 mmol/L; 95% CI −0.24, −0.04; *p* = 0.005 and −0.20 mmol/L; 95% CI −0.33, −0.06; *p* = 0.004, respectively) (Table 2). This was only significant following dinner in the per protocol analyses (*p* = 0.029) (Table S1).

#### 3.5.2 2‐h AUC

The overall and dinner 2‐h AUCs were significantly lower in the active compared with the control intervention (mean difference active vs. control: overall −20.1 mmol/L × min; 95% CI −34.1, −6.1; *p* = 0.005 and dinner −26.1 mmol/L × min; 95% CI −43.4, −8.9; *p* = 0.003) (Table 2). These results were also significant in the per protocol analysis (*p* = 0.034 and *p* = 0.016 for overall and dinner time, respectively) (Table S1).

## 4 DISCUSSION

We previously reported that cruciferous vegetable intake (300 g/day) resulted in a clinically significant reduction in systolic blood pressure and triglycerides compared to root/squash vegetables.^15^ In this secondary outcome analysis, we compared the relative contribution of vegetable types (cruciferous vegetables vs. root/squash vegetables) to glycaemic control. We found that cruciferous vegetable intake resulted in a statistically significant reduction in glycaemic variability (%CV) and PPGR compared to root/squash vegetables. Public health recommendations largely focus on increasing overall vegetable intake. Despite this, vegetable intake remains consistently low,^21^ with cruciferous vegetables among the lowest consumed vegetables.^22^ Recommendations may have greater impact if more emphasis is given to increasing cruciferous vegetables, especially in at‐risk populations.

Glycaemic variability and PPGR have important clinical implications. Glucose fluctuations have been associated with markers of oxidative stress, which may result in poor clinical outcomes and increased cardiovascular events in those with and without diabetes.^23^, ^24^ For a non‐obese, non‐diabetic population, a normal range for glycaemic variability (%CV) is reported as 12–18%,^23^ with stable glycemia in diabetes typically indicated below 36%.^25^ In our study, the results from the active and control intervention groups (18.9% and 20.8%, respectively) were comparable to published results,^23^, ^26^ with improvements in our active intervention approaching the normal range (12%–18%).^23^ Glycemia is typically tightly maintained by physiological mechanisms in healthy individuals; therefore, demonstrating a significant effect in our population without diabetes is an important finding. As these participants did not have diabetes, it is not surprising that there was no difference in mean continuous glucose between interventions. Given the relatively good glycaemic control of our study population, increasing cruciferous vegetable intake in the diet in a population with dysregulated glycaemic control could potentially have a larger effect size on glycaemic control. Similar to %CV, there was a statistically significant reduction in 2‐h AUC in the active compared to the control intervention, driven by the dinner PPGR. As a measure of PPGR, there is no set range for 2‐h AUC in adults without diabetes, and further research is needed to determine a target AUC range for healthy individuals. However, it is important to minimize glycaemic variability and aim for a flatter response curve and lower AUC.^27^

There is a paucity of clinical research investigating the effect of cruciferous vegetable consumption on glycaemic control. To the best of our knowledge, our study is the first study to compare the relative contribution of vegetable types (cruciferous vegetables vs. root/squash vegetables) through the diet on glycaemic outcomes in adults without diabetes. Previous studies have varied in numerous elements, such as the use of vegetables or extracts (e.g., sulforaphane), processing, duration, and population.^11^, ^12^, ^13^ Limited studies using whole‐food cruciferous vegetables have been conducted investigating the benefits of these vegetables on varying measures of glycaemic control, and available research has included a higher vegetable dose and a different population to our study. Increased vegetable intake (500 g/day) resulted in improvements in glycaemic control in a three‐arm 12‐week randomized, controlled parallel trial (*n* = 92 with type 2 diabetes); however, the greatest benefit to fasting glucose and incremental AUC was seen following bitter and strong‐tasting vegetables (e.g., kale, cabbage), which are higher in glucosinolates.^12^ Studies using extracts, resulting in concentrated glucosinolates and isothiocyanates, have also seen positive results on glycaemic control. In a randomized, double‐blind, placebo‐controlled study (*n* = 81 with type 2 diabetes), a 4‐week intervention with 10 g/day broccoli sprout powder (225 μmol sulforaphane) resulted in improved fasting serum insulin and insulin resistance by 18.2% and 14.2%, respectively.^11^ Positive results were also seen in a randomized, double‐blind, placebo‐controlled 12‐week study (*n* = 97 with type 2 diabetes), which found significantly reduced HbA1c following daily consumption of broccoli sprout extract (contains concentrated sulforaphane).^13^ After the broccoli sprout extract intervention, obese participants with dysregulated type 2 diabetes (BMI >30 kg/m^2^, HbA1c >50 mmol/mol 6.7%) had improved fasting glucose (placebo: 8.9 mM, BSE: 8.2 mM; *p* = 0.036) and HbA1c (placebo: 57 mmol/mol 7.38%, BSE: 53 mmol/mol 7.04%; *p* = 0.034) compared to placebo.^13^

The mechanisms by which cruciferous vegetables may result in improved glycaemic outcomes are not well understood. When consumed raw, the mechanical processes of cutting and chewing result in the breakdown of plant tissues, leading to the hydrolyzation of glucosinolates (e.g., glucoraphanin) by the enzyme myrosinase to derivatives such as isothiocyanates and indoles.^28^ Cooking before eating, as was the case in this study, can result in a degradation of myrosinase.^28^ As a result, intact glucosinolates are consumed and hydrolysed into derivatives by intestinal bacteria in the colon.^28^ Glucosinolates and their derivatives, such as the isothiocyanate sulforaphane, are believed to exert their effect on glycemia via activation of the Nrf2 system and associated inhibition of the NF‐ĸB pathway,^29^ with antioxidant and anti‐inflammatory effects.^30^, ^31^ Importantly, we have previously reported that plasma sulforaphane levels increased significantly following the active intervention (mean difference active vs. control: 0.15 ng/mL; 95% CI 0.06, 0.23; *p* = 0.001),^15^ reinforcing this as a potential mechanism for the improvement in glycaemic control seen in the active cruciferous vegetable intervention, compared to the control. Although we have previously reported that we did not see a significant effect on markers of oxidative stress or inflammation between interventions (plasma F_2_‐isoprostanes, serum high‐sensitivity C‐reactive protein, high sensitivity interleukin‐6), we were likely underpowered to detect a significant effect.^15^ Further, we were unable to measure all possible biomarkers of oxidative stress and inflammation. In the VESSEL Study, increased cruciferous vegetable intake also resulted in improvement in other components of metabolic syndrome (i.e., blood pressure, triglycerides).^15^ These conditions are interrelated, with poor glycaemic control and insulin resistance linked with hypertension^32^ and elevated triglycerides.^33^ Although we saw significant differences in glycaemic variability and PPGR, this was not observed for mean continuous glucose over the intervention period. This is not surprising given that our cohort is a non‐diabetic population. However, this could also indicate there are meal and time‐specific effects of cruciferous vegetable intake that require further investigation. Given that our study population did not have diabetes, further exploration into the impact of cruciferous vegetable intake throughout the day should be undertaken in at‐risk populations, such as those with impaired glycaemic control. Whilst the aforementioned research focuses on the glycaemic benefits of glucosinolates, cruciferous vegetables do not contain these bioactive compounds in isolation. It is important to note that vitamin K and nitrate, for example, are present in cruciferous vegetables in greater amounts than root/squash vegetables and have also been associated with a reduced risk of type 2 diabetes and antidiabetic effects, respectively.^34^, ^35^

Lifestyle changes, not limited to cruciferous vegetable intake, may also impact glycaemic control. In this study, participants consumed fewer calories and lost weight following both interventions.^15^ It is important to note that although both interventions resulted in statistically significant weight loss, no significant difference was observed when comparing interventions.^15^ Therefore, this indicates that the benefit seen is likely independent of weight loss. Furthermore, there were no significant differences in physical activity levels between interventions, which is another factor associated with glycaemic control.^36^

Our study has multiple strengths. First, this was a crossover study with an appropriate wash‐out period, with no carryover effect identified between interventions. This design mitigates participant differences, as each acts as their own control. Second, continuous glucose monitoring allowed for increased study power and the investigation of differences at multiple time points. Third, participants were blinded to their glucose data, reducing the possibility of observation bias and changes to their dietary behaviours. Lastly, as mentioned above, diet and lifestyle factors remained consistent across both intervention periods, indicating that the benefits seen are a result of the dietary intervention.

Our study also has limitations. This study can be considered short‐term intake. Therefore, long‐term benefits from glycaemic control are an extrapolation based on short‐term intake. A longer‐term study is needed to determine whether these benefits are maintained over an extended period. We were unable to reach the desired sample size due to the COVID‐19 pandemic.^15^ Despite this, we were still able to detect significant differences in glycaemic variability, 2‐h average glucose, and AUC between interventions. Further, as the majority of our study population were female (16/18), this may limit the generalizability of our results due to sex differences in glucose homeostasis.^37^ Participants also had significant weight loss during both interventions. However, there was no significant difference in weight loss between interventions, and weight loss was slightly greater following the control. This indicates that the different benefits observed in glucose responses between interventions were unlikely to be related to the reductions in weight seen. However, future studies could explore providing a background diet with personalized set caloric intake targets to mitigate energy differences. Due to the distinctive colour/taste differences between vegetables, we were also unable to effectively blind participants to each soup. However, participants were not informed which soup was considered the active/control, nor were they told what vegetables were included. In future studies, the distinctive differences in the active and control vegetables used could be masked by adding these vegetables into mixed meals (i.e., providing a meal that incorporates blended/pureed vegetable with identical seasonings and base ingredients to minimize both colour and taste differences). This could also provide greater variety in study meals instead of the need for twice daily soups. Further, this provides an avenue to incorporate study vegetables into other mealtimes throughout the day (e.g., breakfast and snacks) and reduce variation in other dietary factors that may affect glycaemic outcomes. Given the distinctly different taste of both soups, participant preference could have influenced the speed at which each was consumed, which was not measured in this study. Therefore, possible improvements in blinding mentioned above may result in uniform consumption across interventions. The vegetables used have distinctly different macronutrient content. We added potato starch to the cruciferous vegetable soup to increase the carbohydrate content to match as closely as possible to the macronutrient composition of the root and squash vegetable soup, taking into account the energy provided. This was a difficult task to balance, as increasing the carbohydrate content also increases the protein content. As expected, matching the macronutrient content of the soups could not be exact. Each active soup resulted in 12 lower kilojoules, 4 g lower carbohydrates, 3 g higher protein, 0.2 g higher fat, and 1.5 g higher fibre compared to each control soup. Although these small differences are unlikely to have a substantial effect on our outcomes of interest, we cannot rule out a contribution to the observed effect. Previous studies investigating the effect of carbohydrates, protein, and fat on glycaemic control have required greater differences in these nutrients (i.e., at least 12.5 g, 20 g, and 30 g, respectively) to see an effect on glycaemic control over 2 h.^38^, ^39^, ^40^, ^41^, ^42^, ^43^ Lastly, whilst it has previously been shown that cruciferous vegetable sprouts can improve fasting insulin and insulin resistance in participants with type 2 diabetes,^11^ we have not measured fasting insulin due to a lack of power and the cost of insulin measurements. Future studies should also investigate the effect of cruciferous vegetable consumption on insulin, as this is considered another key factor in glycaemic control.

In summary, consumption of 300 g/day of cruciferous vegetables by middle‐aged and older adults without diabetes who had mildly elevated blood pressure resulted in a significant reduction in glycaemic variability and PPGR compared to root/squash vegetables. As optimal glycaemic control is important in the prevention and management of type 2 diabetes and subsequent CVD risk, targeted recommendations focusing on increasing cruciferous vegetable intake in at‐risk populations may be warranted. Future longer‐term studies are needed to determine whether this benefit is maintained long term.

## AUTHOR CONTRIBUTIONS

L.C.B., R.M., and J.M.H. designed the research. E.L.C. conducted the study with assistance from A.H.L., S.K.G., C.J.S., C.P.B., J.R.L., J.M.H., and L.C.B. Laboratory analysis was conducted by L.G.W. and A.S. E.L.C. performed the statistical analysis in consultation with R.J.W., J.M.H., and L.C.B. E.L.C. wrote the original draft manuscript in consultation and with editing from L.C.B. E.L.C. and L.C.B. had primary responsibility for the final content. E.L.C., A.H.L., R.J.W., A.S., L.G.W., R.M., A.P.J., C.J.S., S.K.G., C.P.B., J.R.L., J.M.H., and L.C.B. critically revised the manuscript, provided an intellectual contribution to the interpretation of results, and read and approved the final manuscript.

## FUNDING INFORMATION

This research was funded by Edith Cowan University Early Career Researcher Grant 2019 (grant number G1004405) and Department of Health Western Australia Near Miss Merit Awards 2019 (grant number G1004519). ELC is supported by an Australian Government Research Training Program Scholarship at Edith Cowan University. CPB is supported by a Royal Perth Hospital Research Foundation (ID: CAF 127/2020) and the Western Australian Future Health Research and Innovation Fund (ID: WANMA2023Ideas/3). LCB is supported by a National Health and Medical Research Council of Australia (ID: 1172987) and a National Heart Foundation of Australia Post‐Doctoral Research Fellowship (ID: 102498). The funding sources were not involved in the design of the study; the collection, analysis, and interpretation of data; writing the report; and did not impose any restrictions regarding the publication of the report.

## CONFLICT OF INTEREST STATEMENT

The authors declare that they have no competing interests.

## PEER REVIEW

The peer review history for this article is available at https://www.webofscience.com/api/gateway/wos/peer‐review/10.1111/dom.16467.

## Supporting information


**Data S1.** Supporting information.

## Data Availability

The datasets used and analyzed during the current study are available from the corresponding author on reasonable request.
