# Supplementary material for: Cruciferous vegetables improve glycaemic control compared to root/squash vegetables in a randomized, controlled, crossover trial: The VEgetableS for vaScular hEaLth (VESSEL) study
Source: Diabetes Obes Metab. 2025 May 15;27(8):4300–10. doi: 10.1111/dom.16467 (PMC12232361; doi:10.1111/dom.16467)
Supplement: Supplementary file 1 — Data S1. Supporting information. [file DOM-27-4300-s001.docx]

Supplementary appendix

**Cruciferous vegetables improve glycemic control compared to root/squash vegetables in a randomized, controlled, crossover trial: the VEgetableS for vaScular hEaLth (VESSEL) study**

Authors: Emma L. Connolly^1^, Alex H. Liu^1^, Richard J. Woodman^2^, Armaghan Shafaei^3^, Lisa G. Wood^4^, Richard Mithen^5^, Anthony P James^6^, Carl J. Schultz^7,8^, Seng Khee Gan^7,9^, Catherine P. Bondonno^1,7^, Joshua R. Lewis^1,7^, Jonathan M. Hodgson^1,7^, Lauren C. Blekkenhorst^1,7^

Affiliations:

^1^Nutrition and Health Innovation Research Institute, School of Medical and Health Sciences, Edith Cowan University, Joondalup, Western Australia, Australia.

^2^Flinders Health and Medical Research Institute, Flinders University, Adelaide, South Australia, Australia

^3^Centre for Integrative Metabolomics and Computational Biology, School of Science, Edith Cowan University, Joondalup, Western Australia, Australia

^4^School of Biomedical Science and Pharmacy, University of Newcastle, Callaghan, New South Wales, Australia

^5^Liggins Institute, University of Auckland, Auckland, New Zealand

^6^Curtin School of Population Health, Curtin University, Perth, Western Australia, Australia

^7^Medical School, University of Western Australia, Perth, Western Australia, Australia

^8^Department of Cardiology, Royal Perth Hospital, Perth, Western Australia, Australia

^9^Department of Endocrinology and Diabetes, Royal Perth Hospital, Perth, Western Australia, Australia

**Supplementary Table 1. Measurements of glycemic control by intervention and between intervention differences following the per-protocol analysis**

| **Outcome** | **Control** | **Active** | **Mean difference active vs. control (95% CI)** |
| --- | --- | --- | --- |
| **Continuous glucose**, mmol/L | 5.30 ± 0.91 | 5.39 ± 0.80 | 0.09 (-0.01, 0.20)  P = 0.092 |
| **Glycemic variability**, %CV | 21.0 ± 5.8 | 19.1 ± 5.3 | -1.94 (-2.81, -1.07)  P < 0.001 |
| **2-hour mean glucose^†^**, mmol/L |  |  |  |
| Overall | 6.47 ± 0.98 | 6.41 ± 0.89 | -0.09 (-0.19, 0.01)  P = 0.074 |
| Lunch | 6.49 ± 1.02 | 6.49 ± 0.91 | -0.02 (-0.16, 0.13)  P = 0.840 |
| Dinner | 6.57 ± 1.63 | 6.43 ± 1.42 | -0.16 (-0.30, -0.02)  P = 0.029 |
| **2-hour AUC**, mmol/L×min |  |  |  |
| Overall | 790.2 ± 122.9 | 779.5 ± 112.1 | -13.7 (-26.4, -1.0)  P = 0.034 |
| Lunch | 792.7 ± 127.6 | 789.4 ± 112.9 | -4.7 (-23.5, 14.1)  P = 0.623 |
| Dinner | 802.7 ± 114.1 | 782.3 ± 109.2 | -21.8 (-39.5, -4.1)  P = 0.016 |

Abbreviations: AUC, area under the curve; CI, confidence interval; CV, coefficient of variation

Values are presented as mean ± standard deviation. Following the per protocol analysis, 1 participant, 3 participants, and 2 participants were excluded from the analysis of overall, lunch, and dinner, respectively, due to incomplete data.

^†^Mean glucose value calculated from measurements obtained every 15 min for 2 hours following the recorded mealtime.

Assessed for eligibility (n = 76)

Analyzed (n = 18)

- All participants included in the intention to treat analysis

## Enrollment

## Follow-Up

## Allocation

## Analysis

Randomized (n = 21)

Lost to follow-up (n = 0)

Discontinued intervention (withdrew due to unwillingness to follow study protocol) (n = 1)

Allocated to intervention (n = 21)

- Received allocated intervention (n = 19)
- Did not receive allocated intervention (withdrew due to scheduling difficulties) (n = 2)

Excluded (n = 55)

- Did not meet inclusion/exclusion criteria (n = 55)

**Supplemental Figure 1. CONSORT flow diagram**
